# Binding Stoichiometry of a Recombinant Selenophosphate Synthetase with One Synonymic Substitution E197D to a Fluorescent Nucleotide Analog of ATP, TNP-ATP

**DOI:** 10.1155/2013/983565

**Published:** 2013-01-30

**Authors:** Y. V. Preobrazhenskaya, A. I. Stenko, M. V. Shvarts, V. Y. Lugovtsev

**Affiliations:** ^1^Yanka Kupala Grodno State University, Grodno 230023, Belarus; ^2^Food and Drug Administration, Bethesda, MD 20892, USA

## Abstract

The transformation of the strain DH5*α* ^TM^-T1^R^ with plasmid vector pET11a containing the cloned gene of bacterial selenophosphate synthetase (SPS), *selD*, from the *E. coli* BL21-Gold (DE3) strain gives an overproducing strain of SPS with one synonymic substitution, E197D. The transformation efficiency was estimated as 8 × 10^8^ CFU/**μ**g plasmid DNA. 28 mg of highly purified preparation of recombinant SPS capable of binding TNP-ATP was eluted from DEAE-Sephadex column in amount of 15 % from the total soluble protein in crude extract. The fluorescent derivative of ATP, 2′(3′)-*O*-(2,4,6-trinitrophenyl)adenosine-5′-triphosphate (TNP-ATP), was used as a synthetic analog of the substrate for the monitoring and quantitative analysis of the functional activity of SPS. The non-linear regression analysis of the saturation curve of TNP-ATP binding to D197 SPS with GraphPad Prism software fits to a model with 2 distinct binding sites with K_D_s different in order. The SPS existence in a form of tetramer in given reaction conditions, in accordance with the concentration stoichiometry of 4 moles of TNP-ATP to 1 mole of recombinant protein, is being discussed. The tetramer structure was predicted with molecular modelling software YASARA and modelled in vacuum using steepest descent minimization energy method. We hypothesize here the recombinant SPS exists as a dimer in solution with two active sites capable of ATP binding in each subunit.

## 1. Introduction

Selenium may be incorporated into a protein body as an amino acid selenocysteine. Selenophosphate synthetase (EC 2.7.9.3.), a product of *selD* gene in bacteria, catalyzes selenophosphate formation with the help of ATP and Mg^2+^ ions [1]. This reaction appears common for all living organisms and serves to transform selenium into biologically active form—selenophosphate. Then, in bacteria selenoprotein synthesis is performed during a few definite steps. First, selenocysteine-specific tRNA (tRNA^Sec^) is aminoacylated with serine—and this tRNA is unique for this amino acid! It means that selenocysteine has its own tRNA—it is a 21^st^ amino acid in the genetic code. After that, the formated product is easily converted to selenocysteine—tRNA^Sec^ with selenophosphate made at the previous step [2]. Further, selenocysteine formed could be incorporated into protein molecule that is encoded with UGA codon [3]. Thus, in contrary to the other amino acids, the selenocysteine biosynthesis is carried out on its tRNA [4, 5]. In eubacteria, monoselenophosphate could be used as a selenium donor as for incorporation of selenium into the protein body as far as for conversion of thiouridine to selenouridine in tRNA [6]. There are two homologs of SPS in human: SPSI and SPSII. Recently the interesting data were obtained about the involvement of SPSII into cancerogenesis, however, the distinct role the enzyme plays in the malignation process remains obscure. The homolog of SPS from *E. coli* is usually used as a model for studies of SPS function and properties regarding to its high structural similarity—55% [7]. It is supposed to be a 37-kDa monomeric protein, dependent on ATP and Mg^2+^. In the protein structure one can find two glycine-rich highly conservative regions, one in the N-terminus at 14–21 [3] (G1), the other—in the C-terminus [7] (G2). Different investigators considered them as potential sites for ATP-binding, however, nowadays is definitely clear only, that in one of them, G1, Cys17 is a site for selenide ion binding, therefore, Cys17S is a catalytically inactive mutant that retaines only about 12% of its ATPase activity.

Recently, the methods of presteady-state kinetics become more and more common concerning their relatively high velocities, in comparison to steady-state, and much higher specificity, for instance, the method of structural fluorescent probes [8]. When investigating comlpex formation it is worth to follow the fluorescence of a ligand. In this work, the functionally active fraction of SPS was monitored following the complex formation with a fluorescent nucleotide analog for ATP, TNP-ATP.

In the present report we describe the overexpression and purification strategy for preparation of large quantities of soluble recombinant SPS from *E. coli* and its binding stoichiometry to TNP-ATP in order to determine its functional activity.

## 2. Experimental

### 2.1. The Vector Construction for the Expression of Selenophosphate Synthetase in *E. coli*

The plasmid pET11a was used as an expression vector (Stratagene Cat no. 211521) [9]. For the cloning of the full-size gene *selD* from *E. coli *the next primers were synthetized: forward with the NdeI recognition site, CATTGACAGGAGATG**CATATG**AGCG; and reverse, with site recognition for BamHI: TTGAGATTCGTTAATTCTAG**GGATCC**GTTTATT. The size of the amplicon obtained is as long as 1081 base pairs. The polymerase “PfuUltra HotStart High Fidelity DNA Polymerase” (Stratagene, Cat no. 600390) was used for the amplification. The PCR-product was purified from 1% agarose gel with a reagents kit QIAquick Gel Extraction Kit (QIAGEN, Cat no. 28706) following the endonucleases treatment and ligation with the plasmid vector. As a template DNA for amlification in PCR the genomic DNA from *E. coli* of strains DH5*α*-T1 (Invitrogen, Cat no. 12297-016) and BL21-Gold (DE3) (Stratagene, Cat no. 230132) was used. The DNA extraction from the bacterial culture was performed with a kit of reagents “Genomic DNA Kit” (Bio-Rad, Cat no. 732-6340).

Treatment of the plasmid vector and insertion segment (the PCR product) with the BamHI and NdeI endonucleases as well as subsequent ligation with the T4 DNA Ligase was performed in compliance with the manufacturing company (New England Biolabs, USA) guidelines. Nucleotide sequence of the insertion segment was controlled with a direct sequencing of the plasmid samples (FDA sequencing core, Bethesda, USA). For that, the plasmid DNA was isolated from the bacterial culture with the “QIAprep Spin Miniprep Kit” kit (QIAGEN, Cat no. 27106) and purified with the ethanol precipitation method as stated in [10]. Quantitative evaluation of the amplicon was monitored spectrophotometrically at A_260_. According to [11], the DNA concentration amounts to 50 *μ*g/mL at A_260_ = 1.

### 2.2. Competent Cells of BL-21-Gold (DE3) *E. coli* and Transformation

The *selD* gene was cloned into pET11a plasmid vector (Novagen, USA). The cells of *E. coli* of strain BL-21-Gold (DE3) Competent Cells (Stratagene, Cat no. 230132) were used for obtaining the recombinant SPS preparation. The recombinant vector containing *selD* gene was transformed according to the manufacturer recommendations (Stratagene). In some cases, chemically competent cells were prepared by Mandel and Higo method using CaCl_2_ [12]. All the reagents used were of analytical grade of purity, except the mentioned solely. The SOC medium used for transformation was sterilized after adding glucose passing through Corning filters, 0.2 *μ*m (Germany). For selection of positive clones of the transformed cells, expressing the recombinant SPS, the medium containing 100 *μ*M of ampicillin was applied. The effectivity of transformation was calculated by the method of the strain producer, DH5*α*-T1 (Invitrogen) [13].

### 2.3. Expression, Isolation, and Purification of a Recombinant Selenophosphate Synthetase (SPS)

To receive bacterial biomass, the selected cells containing the recombinant plasmid were grown at 150 rpm, in shaking 500-mL flasks containing LB-medium with MgCl_2_ × 6H_2_O 0.2 mg/mL and ampicillin of 100 *μ*g/mL. Induction of overexpression of the plasmid pET11aSelD was conducted with injection of IPTG (Sigma, USA) (0.5 mM final) at A_600_ = 0.6 and after that the culture was grown for 3-4 hours. To determine the optimal time for protein production aliquotes of the cultural medium were taken after 1, 2, 3, and 4 hours of expression starting. The cultural biomass of BL-21 *E. coli* cells containing the recombinant plasmid was centrifuged for 10 min at 8000 g to separate cells from cultural medium, resuspended in the buffer for SPS extraction, developed by us earlier [14] (5 mM MgCl_2_, 1 mM EDTA, 2 mM DTT, in the medium of 0.1 M Tris-HCl, pH 7.5), and disrupted with ultrasonic desintegrater UD-20 (Poland) at maximal regime for 5 min on ice. After that it was centrifugated at 8000 g for 20 min to remove cell debris. The prepared crude cell extract was lightened with centrifugation at 2500 rpm for 5 min at 4°C. The soluble protein fraction then underwent ammonium sulfate precipitation carried out on ice, for short, the maximal amount of SPS was found in the fraction when 55% of ammonium sulfate was added and then centrifuged at 6000 g for 15 min. The precipitate was resuspended in 5 mL of 0.1 M Tris-HCl, 2 mM DTT, pH 7.5 and dialyzed against 1 L of 0.01 M Tris-HCl (pH 7.5), containing 2 mM DTT and 0.5 mM EDTA, at 4°C during 24 hours. Further, to all protein-containing fractions during purification 0.5 mM EDTA was added to keep protein soluble. The next step in recombinant protein purification was anion-exchange chromatography that was performed as developed earlier [15] and modified by us recently [16]. The modifications were the next: change of the resin for Sephadex A-50 that allowed doing ion-exchange only once, without rechromatography, and differences in buffer components. The column with the carrier DEAE-Sephadex A-50 (parameters 50 × 1, 7 cm) was equilibrated with the same buffer used for the dialysis (above), the pH used for loading onto the carrier was not changed in comparision to wild-type protein as far as Glu→Asp substitution could not influence on isoelectric point of the protein. The protein elution from the column was performed using step-gradient of KCl.

The presence of SPS in the fractions after the column was monitored on maximum shift of the fluorescence emission and change of fluorescence intencity of TNP-ATP, a fluorescent nucleotide analog of ATP. The purity of the protein preparation was checked with the method of SDS-PAAG electrophoresis, as molecular weight markers a protein ladder for SDS-PAAG electrophoresis {“SeeBlue Plus Pre-Stained Standard” (Invitrogen, Cat no. LC5925) was used.

The obtained after the stage of anion exchange chromatography protein preparation was applied for further investigations of the complex formation between SPS and a low-weight fluorescent ligand, TNP-ATP.

The protein concentration was determined spectrophotometrically at A_280_ and then calculated using the molar extinction coefficient 16.1 M^−1^ × cm^−1^ [17].

### 2.4. Investigation of Complex Formation of Recombinant SPS with a Fluorescent Analog of ATP, TNP-ATP

A highly-purified preparation of the enzyme after the stage of anion-exchange chromatography on DEAE-Sephadex A-50 and a fluorescent nucleotide analog of ATP, TNP-ATP (Molecular Probes, Cat no. T7602), were used for protein-ligand complex investigations. For fluorescence spectra detection of the ligand and the protein-ligand complex a spectrofluorometer CM2203 (Solar, Belarus) from the Laboratory of Molecular Spectroscopy of Grodno State University was applied. The binding reaction was conducted in Tris-HCl buffer (25 mM, pH 7.0), containing 100 mM NaCl, 30 mM KCl, 10 mM MgCl_2_ under the method, developed by us earlier [15, 16]. Scanning of emission spectra of the ligand and the protein-ligand complex was performed from 500 to 600 nm and excitation wavelength of 408 nm was used. The fluorescence intensity enhancement and a spectral shift to the red upon addition of the protein probe to the fluorescent dye, TNP-ATP, was considered as a positive answer of the fluorescent probe to the presence of active SPS in the fractions during the purification process.

The fluorescence titration of the recombinant SPS with TNP-ATP was performed in the range of 2–16 *μ*M of TNP-ATP. The difference between the fluorescence intensity emission of the dye itself and the complex dye-protein was determined as fluorescence intensity enhancement. Upon the difference on fluorescence intensity of the dye and the complex dye-protein without the fluorescence emission for the protein itself the amount of fluorescence emission for the complex was calculated. For binding stoichiometry estimation the plot of fluorescence intensity of the complex versus TNP-ATP amount added to the probe of protein was built (a so-called saturation curve). The data of fluorescence titration were presented graphically and analyzed using GraphPadPrism 5.0 analytical software (GraphPad Software, USA).

The development of fluorescent spectra was performed with Origin 6.0 program.

## 3. Results

### 3.1. Vector Construction on the Base of Plasmid pET11a

**Figure 1:**
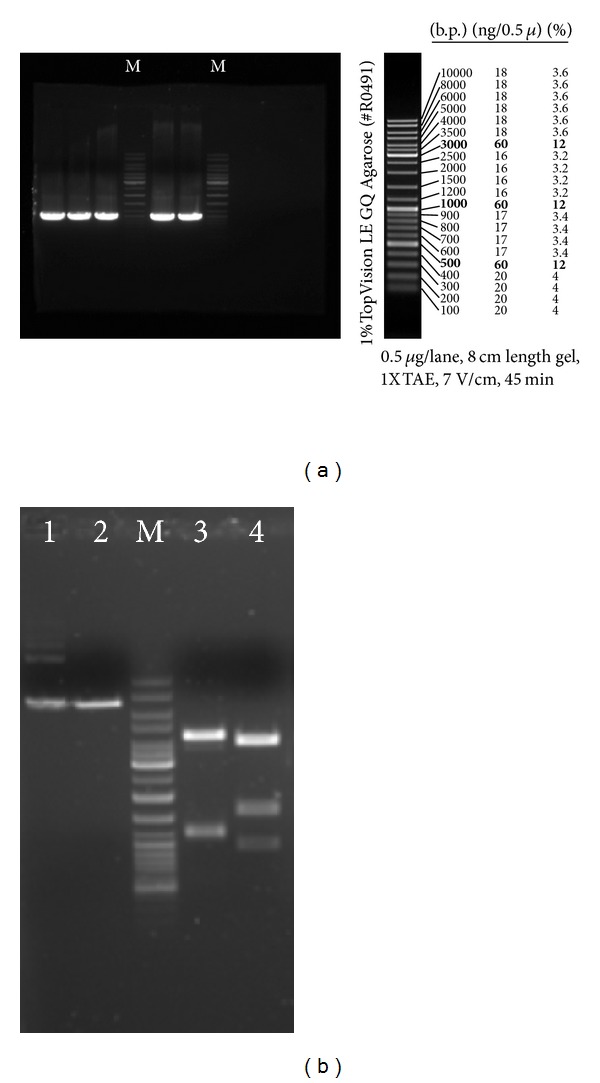
(a) PCR-product of *selD* amplification (size—1081 b.p.; 5 × 40 *μ*L). MM—DNA marker GeneRuler Ladder Mix (Fermentas, Cat #SM0333). (b) The restriction profile of a plasmid with correct orientation of *selD* gene: lane 1, full plasmid; lane 2, linear plasmid after digestion with SalI; lane 3, plasmid after digestion with (a), fragments size: 51 + 1163 + 1252 + 4215 b.p.; lane 4, plasmid after digestion with BspHI, fragments size: 105 + 1008 + 1652 + 3926 b.p. (fragments of small size 51 b.p. and 105 b.p. are not seen in the gel).

The good yield of PCR-product was observed under the next reaction conditions: 2,5 U of the enzyme and 169.5 ng/*μ*L DNA was taken for the reaction, the one was performed during 35 cycles, elongation time = 5′30′′, in a total volume of 100 *μ*L. The yield of PCR-product was 13.468 *μ*g. A_260_/A_280_ for the product received is equal to 1.88 that shows the purity of the recombinant DNA, according to [11]. After treatment of the insertion segment (PCR product) and vector (plasmid) with restriction endonucleases, DNA fragments (insertion segment + vehicle) were ligated, and the plasmid vectors obtained were used for the transformation of competent cells to amplificate the plasmids in selective medium (with ampicillin). Samples of transformed cell mini-preparations were analyzed for presence of plasmids with correct insertion segment via the restriction analysis (using the ApoI or PvuII enzymes; Figure 1(b)) and sequencing.

**Figure 2:**
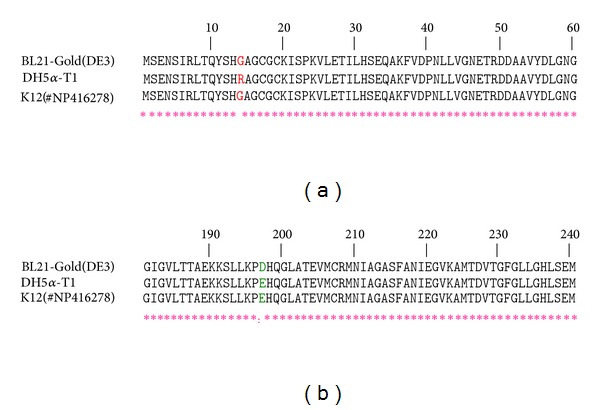
Alignment of amino acid sequences. (a) Amino acid substitution in position 14 in the strain DH5*α*-T1. (b) Amino acid substitution in position 197 in the strain BL21 Gold (DE3).

As far as the *selD* gene sequences from the *E. coli* DH5*α*-T1 and BL21-Gold (DE3) strains are not presented in the gene bank (GenBank, NCBI), comparison of the samples obtained was made with a sequence of the *selD* gene from the *E. coli* K12 strain (GenBank Accession no. NP416278). The alignment showed that in the amino acid sequence derived from the *selD *gene, isolated from the strain DH5*α*-T1, at 14 position there is Arg, when we have Gly in the same position of AA sequence derived from the *selD* gene that belongs to strain K12 (GenBank Accession no. NP416278) (Figure 2(a)). At the same time, *selD*, isolated from the strain BL21-Gold (DE3), encodes Gly in position 14, but differs from straines K12 and DH5*α*-T1 in position 197: SPS from strain BL21-Gold (DE3) has Asp residue in 197th position, when the other two strains have Glu (Figure 2(b)). For the futher isolation and purification of the SPS the plasmid with the gene from the strain BL21-Gold (DE3), taking into consideration that the difference E197D could be named synonymic and perhaps has minimal influence on the functional properties of the protein.

The selected clones with the transformed cells with the plasmid containing the gene *selD* in correct orientation were amplified to receive preparative amounts of the recombinant SPS preparation.

### 3.2. Transformation of *E. coli* Competent Cells with the Recombinant Plasmid pET11aSelD

While transforming competent cells of the DH5*α*-T1  strain with the recombinant plasmid pET11aSelD that contained the cloned SPS gene from *E. coli* BL-21, an intensive culture growth was observed after plating it on LB agar with ampicillin (100 *μ*g/mL). Transformation efficiency exceeded 100 CFU per 1 plate which corresponds to approximately 8·10^8^ CFU/*μ*g of plasmid DNA. Intensive growth of colonies up to 3-4 mm in size was already observed in 8-9 hrs (at 37°C).

### 3.3. Expression, Isolation, and Purification of a Recombinant Selenophosphate Synthetase (SPS)

**Figure 3:**
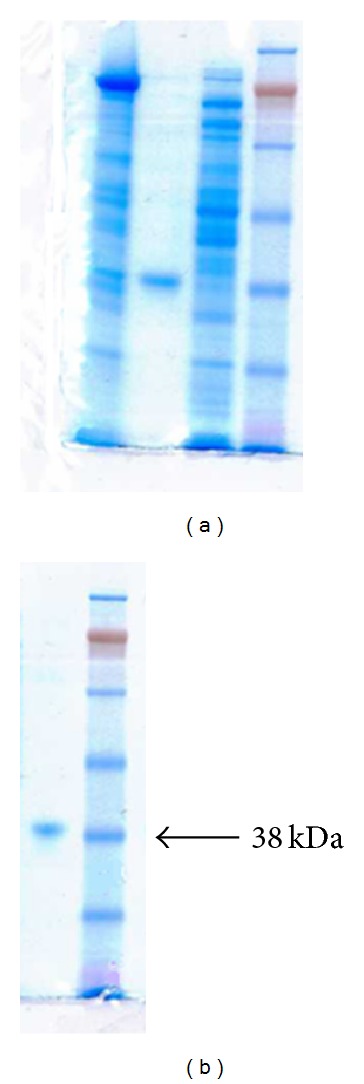
SDS-PAAG-electrophoresis of the recombinant SPS preparation. From left to right: (a) Raw extract, supernatant; highly-purified SPS (M.W. = 37.5 kDa); raw extract, pellet; a protein ladder of markers (SeeBlue Plus Pre-Stained Standard; Invitrogen, Cat # LC5925), (b)—a highly-purified preparation of SPS after DEAE-Sephadex A-50, markers. Stained with Brilliant Blue G.

**Figure 4:**
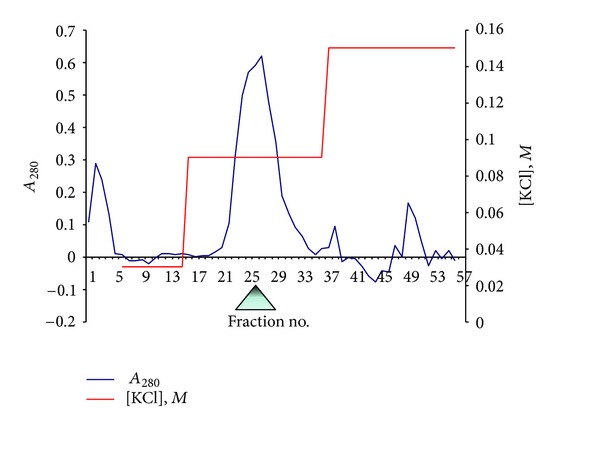
Anion-exchange chromatography on DEAE-Sephadex A-50 of SPS E197D preparation. △—the base of the triangle shows the active fractions of SPS, able to bind TNP-ATP.

The results that demonstrate overexpression of a recombinant SPS with a single synonymous replacement E197D are represented in Table 1. As can be seen, the induction of the protein plasmid expression with IPTG promoted an increase in the *selD* gene product's yield as much as approximately 3.5 times. It has also been experimentally established that the enzyme content after the IPTG injection is maximized in 3-4 hours. The temperature induction of the overexpression described in [14] was not carried out. Protein electrophoresis of a cell lysate of the BL21 (DE3) producer strain transformed with the pET11aSelD plasmid has shown that as a result of the IPTG induction, the SPS gene, *selD*, successfully overexpresses. At that, the main part of the protein sought is situated in soluble fraction (Figure 3(a)). After fractioning with ammonium sulphate, SPS was contained in a precipitate settled within the fraction of 0–55% saturation. The protein sought is completely precipitated within the given range of the salt concentration. Under this procedure, about 18.7% of total protein is removed along with the supernatant fraction. At the stage of anion-exchange chromatography, SPS is firmly bound to the DEAE-Sephadex A-50 anion-exchange carrier and effectively eluated by the KCl solution at the salt concentration of 0.09 M (Figure 4). The purity of the preparation obtained (fraction pool 23–29, Figure 3) was confirmed by an electrophoresis in SDS-PAAG (Figure 3(b)). Monitoring of the functional activity of the purified SPS preparation fractions was conducted by a complex formation with the TNP-ATP ligand, according to our procedure developed earlier [15, 16].

The procedure described above allows obtaining 28 mg of the SPS preparation of a high purity extent from 6 g of cell biomass that amounts to 15% of a total soluble protein of the crude cell extract.

### 3.4. Control of the SPS Preparation Presence by Formation of a Complex between TNP-ATP and SPS in the Fractions after the Purification

**Figure 5:**
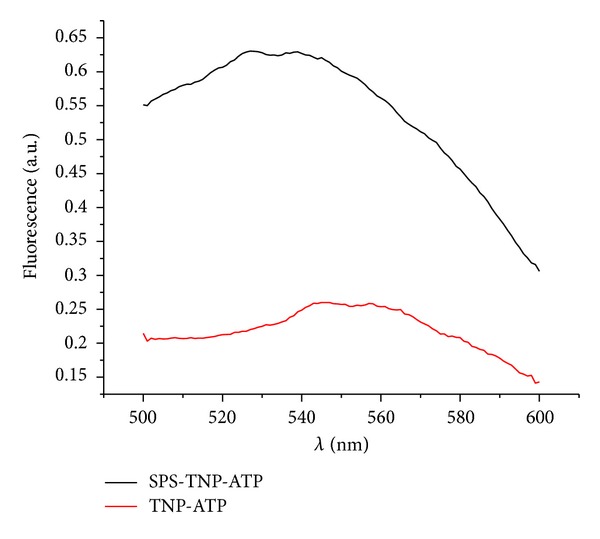
Fluorescence spectra of TNP-ATP** (**4 *μ*M**)** and of the SPS-TNP-ATPcomplex **(**[SPS] = 1.08 *μ*M**)**.

During the TNP-ATP binding with a hydrophobic active center of an ATP-binding enzyme, both an increase in fluorescence intensity and a shift of the maximum fluorescence emission to the short-wave region is observed that allows to control the complex formation [18] (Figure 5). After DEAE-Sepharose, there were no other ATP-binding proteins in the SPS preparation except SPS itself [14] that was verified by the SDS-PAAG electrophoresis in our case (see Figure 3(a)). Regarding to that, the reaction of the TNP-ATP-SPS complex formation seems to be strongly specific after the anion-exchange chromatography stage.

In the samples that did not contain protein, the fluorescence intensity depended linearly on the TNP-ATP fluorescent probe concentration (data not shown). Data regarding the complex formation (see Figure 5) are represented in the form of spectra: the lower spectrum corresponds to a protein-free TNP-ATP, the upper one—to TNP-ATP with a protein. As can be seen, this probe is of a faint fluorescence with maximum at 555 nm in water solutions. When adding the protein (SPS) to TNP-ATP, along with increasing (~3 times) in fluorescence intensity, a short-wave spectral shift for ~10–15 nm is observed that allows monitoring presence of the functionally active enzyme in the preparation fractions during the purification process (the analysis of fractions after the anion-exchange chromatography with DEAE-Sephadex A-50).

### 3.5. Fluorescent Titration of SPS with Increasing Concentrations of TNP-ATP

The fluorescent titration of SPS (1.08 *μ*M) with increasing concentrations of TNP-ATP was performed at the range of concentrations (2–16 *μ*M), when the calibration curve for free TNP-ATP is linear (not shown). The titration data was developed with analytical software GraphPadPrism 5.0 (USA).

The saturation curve of the recombinant SPS with a fluorescent nucleotide analog of ATP, TNP-ATP, reaches plateau that allows using this fluorescent probe for quantitative analysis of binding parameters. The analysis of the form of the curve with nonlinear regression method gives a nonspecific binding with two distinct binding sites for TNP-ATP, the K_D_s of which differ in a factor of ten (see the instruction manual of the manufacturer GraphPad Software, Inc.), *R* ^2^  for  this  curve = 0.9861. Slight decrease in fluorescence intensity after the curve reaches maximum could evidence about just a few degradation of the ligand [19]. One should point out that in given reaction conditions the system “protein-ligand” reaches saturation at 4 *μ*M of TNP-ATP yet.

Adding of a natural substrate, ATP, to the complex of SPS with TNP-ATP reveals a competitive displacement of TNP-ATP with the increasing ATP concentration. At about concentrations, close to those observed in the living cell, ATP displaced TNP-ATP with EC_50_ = 0.45 mM that is very close to k_M_ (Mg-ATP) = 0.5 mM (not shown). It might mean there is a competition between the real substrate, Mg-ATP, and the ligand, TNP-ATP, in the active site of the enzyme. The fluorescent ligand therefore exhibits the competitive inhibitor effect on Mg-ATP [20].

**Figure 6:**
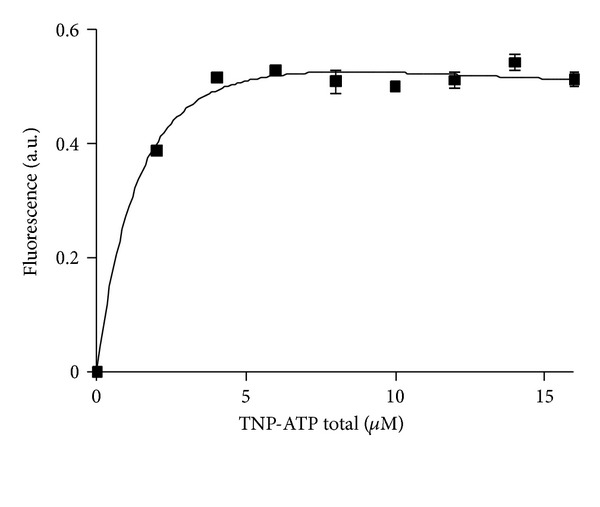
Dependence of the equilibrium concentration of the complex SPS-ligand on the concentration of the ligand (TNP-ATP) ([SPS] = 1.08 *μ*M).

Taking into consideration the competitive inhibition of Mg-ATP with TNP-ATP we used the fluorescent analog to determine the number of active sites for Mg-ATP. As it easily could be seen from the graph (Figure 6), the binding stoichiometry is 1 mole of the protein per 4 moles of TNP-ATP. The other question is oligomeric state of the protein. Taking into consideration that the molar extinction coefficient given was determined at 25 *μ*M concentration of the protein [17] one could assume at such concentration almost all the protein would be associated in dimeric form as far as there is already data about easy dimerization of the members of PurM family to that SPS belongs to, and, in particular, SPS from *Aquifex aeolicus* [21]. Therefore, we conclude 2 moles of a monomer per 4 moles of TNP-ATP and, we have got two moles of TNP-ATP bound to a monomer/subunit. According to our data on competitive displacement, there are two moles of ATP able to bind to a subunit of the recombinant SPS. Regarding to different groups [3, 7], investigators point out two glycine-rich conservative regions in the protein 348-amino acid sequence that could be considered as distinct active sites: one is situated in the N-terminus at 14–21; the other is in the C-terminus. It is likely, 1 molecule of TNP-ATP/ATP binds to each of the proposed active sites.

### 3.6. Experiment of Energy Minimization with Tetramer Structure of SPS

We predicted the tetramer structure of SPS with DEDAL program [22] that during alignment built a tetramer consisting of two dimers that were aligned in N-termini within 55 amino acids the same as in the hexameric structure of *Aquilex aeolicus* [21]. The dimers were taken from Protein Data Base Bank. After that, the structure was relaxed with YASARA program. To remove bumps and correct the covalent geometry, the structure was energy-minimized with the YAMBER3 force field, using a 7.86 Å force cut off and the Particle Mesh Ewald algorithm to treat longrange electrostatic interactions. After removal of conformational stress by a short steepest descent minimization, the procedure continued by simulated annealing until convergence was reached, that is, the energy improved by less than 0.05 kJ/mol per atom during 200 steps. During the procedure the RMSD of the tetramer reduced from 48.0047 till 39.4 Å . The tetramer conserves the central barrel characteristic for the members of PurM family. At last, to check minimum distances between the atoms, the next algorithm (in Python) was applied:

 for *a* in AllAtoms ():
 for *b* in AllAtoms ():
 if distanse(*a*, *b*) < min:
 min⁡ = distanse(*a*, *b*)

The structure of a tetramer modelled is given in (Supplement 1 in Supplementary Material available online at http://dx.doi.org/10.1155/2013/983565).

The central barrel is shown in (Supplement 2 in Supplementary Material available online at http://dx.doi.org/10.1155/2013/983565).

Two chains in a dimer colored in different color shown in Pymol Viewer program are presented in (Supplement 3 in Supplementary Material available online at http://dx.doi.org/10.1155/2013/983565).

## 4. Discussion

Selenophosphate serves as a universal selenium donor in cellular processes both in prokaryotes and eukaryotes, and its formation is catalyzed by selenophosphate synthetase (SPS), a *selD* gene product [23]. However, an attempt to substitute the SPS II gene from mammals for the prokaryotic *selD* gene was not successful: when the SPS II gene was inserted into a *selD*-deficient *E. coli* culture, the culture growth was distinctly inhibited that indicated a lethality of the bacterial gene replacement by a homolog from mammals [3]. Originally, with the purpose of obtaining an organism that produces SPS, we cloned a *selD* gene from the *E. coli* DH5*α*-T1 strain. However, the sequencing has shown that a primary structure supposed for a protein derivative of the *selD* gene for the given strain contains the Arg residue rather than Gly in the 14th position, as shown for the K12 strain (GenBankAccession no. NP416278), MB08 strain which was earlier used for creation of the SPS gene product [24], and BL21-Gold (DE3) strain (present work). In this connection, another plasmid construction was created with the *selD* gene from the *E. coli* BL21-Gold (DE3) strain encoding the Gly residue in the 14th position. However, the *selD* gene from this strain was also dissimilar to those from K12 and DH5*α*-T1 strains encoding the Asp residue in the 197th position, whereas it encoded the Glu residue for other two strains. As far as the Asp and Glu residues are equally ionized within a protein molecule, the plasmid with the *selD* gene from the BL21-Gold (DE3) strain was used for subsequent isolation and purification of SPS.

The *E. coli* DH5*α*-T1 strain transformation with the pET11aSelD plasmid from the *E. coli* BL21-Gold (DE3) strain did not negatively affect the cell viability. An effective growth of colonies transformed by *E. coli* was observed after inoculation of a selective medium. Besides, the induction of the given protein expression using IPTG had also no negative effect on the producing cells [11].

SPS is isolated well from cells, full isolation of the enzyme occurs when extracting with a buffer of the proposed composition. After induction with IPTG, expression level of the cloned *selD* gene in the DH5*α*-T1 strain cells amounted to 15% of the cell protein total amount, at that, the most part of the expression product was in a soluble form. For purifying the recombinant SPS from a soluble fraction of cell proteins, a simple and convenient method has been developed consisting of only two stages: fractionation with ammonium sulphate and anion-exchange chromatography with DEAE-Sephadex A-50. The method allows obtaining a high-purity protein with a yield ~20 mg/L of a bacterial culture. Hydrophobic chromatography is not required because of the adenylate kinase impurities contaminating the preparation by [14] were not found in our SPS preparation, according to the gel electrophoresis (see Figure 4). This is probably bound with the protein solubility increasing because of a replacement by the amino acid with a shorter hydrocarbon chain Glu197Asp. For all that, SPS does not form inclusion bodies, in contrast to the case of inserting the point mutation *selD* gene from the MB08 strain into the BL21(DE3) strain [25].

The quantitative analysis of fluorescent titration data with increasing TNP-ATP concentration showed that at [SPS] = 2 *μ*M the binding curve does not reach saturation [26]. If to decrease the SPS (protein) concentration to ~1 *μ*M, we obtained a saturation curve with the form typical for two-site specific binding. The result obtained for stoichiometry 1 : 4 could be explained with two variants: either we have a tetramer or recombinant SPS represents a dimer at given conditions with two binding sites for ATP on each subunit. Regarding to the data received by the group for *Aquifex aeolicus* that SPS is a dimer [21], the second suggestion might be more appropriate. Based on the data for catalytically inactive mutant, C17S, it seems unreasonable to make conclusions about the active site arrangement as far as it is obvious the protein is in inactive conformation (apo-form).

Thus, in the present work, we showed functional expression of the bacterial SPS with one synonymic substitution and its binding stoichiometry to TNP-ATP, a fluorescent nucleotide analog of ATP. To determine a functional active conformation of the native type of SPS, not a mutant, further investigation of its properties at the reaction conditions should be performed.

## Supplementary Material

The structure of a tetramer modelled is given in (Supplement 1). The central barrel is shown in (Supplement 2). Two chains in a dimer colored in different color shown in Pymol Viewer program are presented in (Supplement 3).

## Figures and Tables

**Table 1:** Purification of the prokaryotic SPS from the *E. coli* DH5*α*-T1 strain containing a plasmid with a cloned *selD* gene from the BL21-Gold (DE3) strain.

Expression	Purification index			
	Cell quantity, raw weight	Protein amount in the raw extract, mg	Amount of the protein loaded onto a column with DEAE-Sephadex, mg	Amount of the protein eluted from the column, mg
Without an inductor	6	75	61	7.8
With an inductor (IPTG)	6	185	150	28
