# Supplementary figures and images for: Binding Stoichiometry of a Recombinant Selenophosphate Synthetase with One Synonymic Substitution E197D to a Fluorescent Nucleotide Analog of ATP, TNP-ATP
Source: J Amino Acids. 2013 Jan 30;2013:983565. doi: 10.1155/2013/983565 (PMC3956282; doi:10.1155/2013/983565)

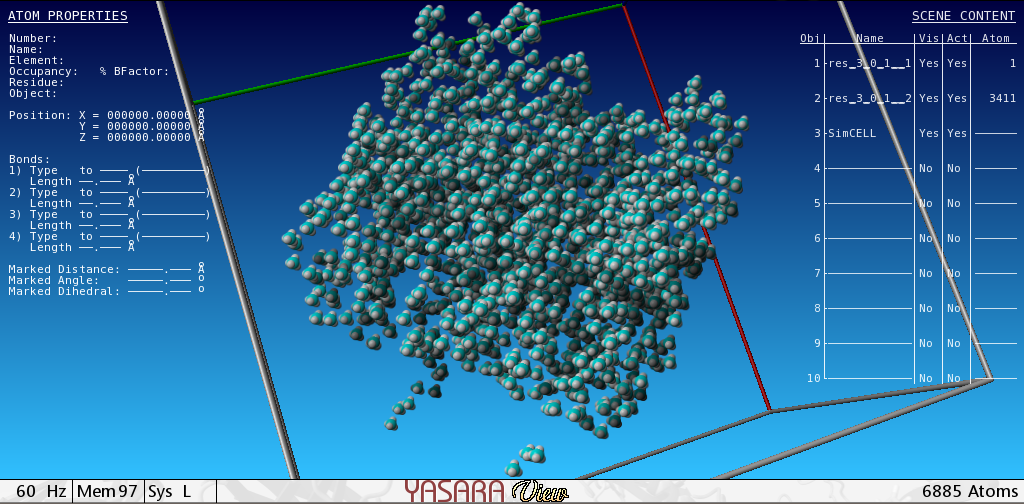

Supplement: Supplementary file 1 — The structure of a tetramer modelled is given in (Supplement 1). The central barrel is shown in (Supplement 2). Two chains in a dimer colored in different color shown in Pymol Viewer program are presented in (Supplement 3). [file 983565.f1.png]

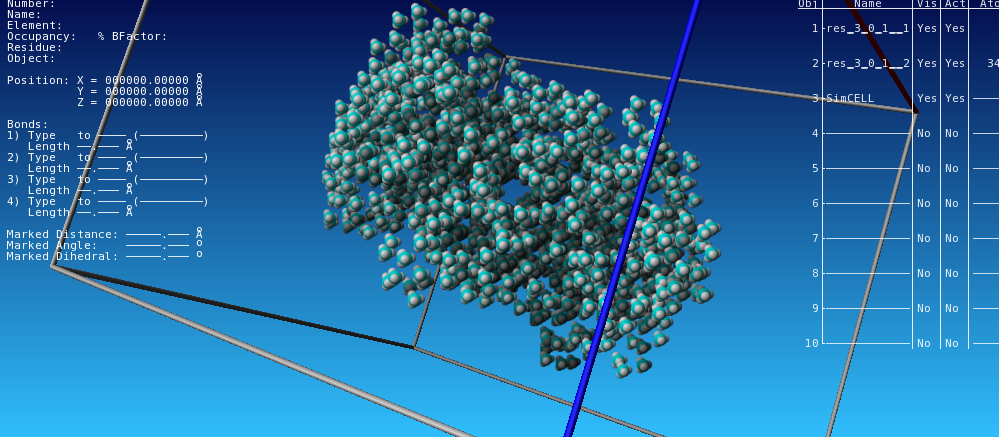

Supplement: Supplementary file 2 [file 983565.f2.png]

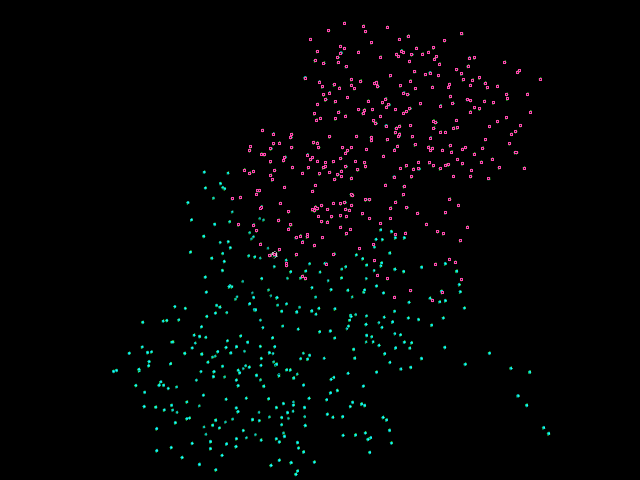

Supplement: Supplementary file 3 [file 983565.f3.png]
